# Mutation of 4-coumarate: coenzyme A ligase 1 gene affects lignin biosynthesis and increases the cell wall digestibility in maize *brown midrib5* mutants

**DOI:** 10.1186/s13068-019-1421-z

**Published:** 2019-04-10

**Authors:** Wangdan Xiong, Zhenying Wu, Yuchen Liu, Yu Li, Kunlong Su, Zetao Bai, Siyi Guo, Zhubing Hu, Zhiming Zhang, Yan Bao, Juan Sun, Guofeng Yang, Chunxiang Fu

**Affiliations:** 1 Key Laboratory of Biofuels, Shandong Provincial Key Laboratory of Energy Genetics, Qingdao Institute of Bioenergy and Bioprocess Technology, Chinese Academy of Sciences, Qingdao, 266101 China; 2 Institute of Plant Stress Biology, State Key Laboratory of Cotton Biology, Department of Biology, Henan University, Kaifeng, 475001 China; 3 Maize Research Institute, Sichuan Agricultural University, Chengdu, 611130 Sichuan China; 4 School of Animal Science and Technology, Qingdao Agricultural University, Qingdao, China

**Keywords:** Maize, *brown midrib*, Cell wall digestibility, Lignin, 4-coumarate: coenzyme A ligase

## Abstract

**Background:**

Maize *brown midrib* (*bm*) mutants associated with impaired lignin biosynthesis are a potential source for the breed of novel germplasms with improved cell wall digestibility. The spontaneous *bm5* mutants had been identified since 2008. However, the gene responsible for the *bm5* locus, and the comprehensive effects of *bm5* mutation on lignin biosynthesis, soluble phenolics accumulation, and cell wall degradation have yet to be elucidated.

**Results:**

The *bm5* locus was identified to encode a major 4-coumarate: coenzyme A ligase (Zm4CL1) through analyzing MutMap-assisted gene mapping data. Two alleles of *Zm4CL1* isolated from *bm5* mutants contained two transposons inserted in the first exon and the second intron, respectively, and consequently, the activities of 4CLs in the crude enzyme extracts from *bm5* midribs were reduced by 51–62% compared with the wild type. Furthermore, five *4CLs* were retrieved from maize genome, and *Zm4CL1* was the most highly expressed one in the lignified tissues. Mutation of *Zm4CL1* mainly impeded the biosynthesis of guaiacyl (G) lignins and increased the level of soluble feruloyl derivatives without impacting maize growth and development. Moreover, both neutral detergent fiber digestibility and saccharification efficiency of cell walls were significantly elevated in the *bm5* mutant.

**Conclusions:**

Zm4CL1 was identified as the *Bm5* gene, since two independent alleles of *Zm4CL1* were associated with the same mutant phenotype. Mutation of *Zm4CL1* mainly affected G lignin biosynthesis and soluble feruloyl derivatives accumulation in maize lignified tissues. The reduced recalcitrance of the *bm5* mutant suggests that *Zm4CL1* is an elite target for cell wall engineering, and genetic manipulation of this gene will facilitate the utilization of crop straw and stover that have to be dealt with for environmental protection.

**Electronic supplementary material:**

The online version of this article (10.1186/s13068-019-1421-z) contains supplementary material, which is available to authorized users.

## Introduction

Plant cell walls are structural supporter and natural barrier in protecting the plants from pathogens and insects, which consist mainly of cellulose, hemicellulose, and lignin [1]. Lignin is a complex and heterogeneous aromatic polymer that is an important component for structural support, water transport, and biotic and abiotic stress defenses during plant growth and development [2]. The biosynthesis of monolignols generates from the general phenylpropanoid metabolism, following by a series of hydroxylation and methylation reactions [2]. It has been reported that lignin content and its subunit composition are closely related to the bioconversion efficiency of the lignocellulosic biomass into fermentable sugars [1, 3]. Moreover, the presence of lignin in cell walls also negatively impacts forage digestibility and pulping efficiency [4].

4-coumarate: coenzyme A ligase (4CL) is an essential enzyme in lignin biosynthetic pathway, converting *p*-coumarate, caffeate, and ferulate into their corresponding CoA esters [2, 5]. The 4CL family is a small and conserved gene family, playing a critical role in the biosynthesis of phenylpropanoid metabolites [6]. The 4CLs can be divided into two major subgroups: one group is mainly responsible for lignin biosynthesis; the other group is involved in flavonoid biosynthesis [6]. In *Arabidopsis*, three *4CL* isoforms, namely *At4CL1*, *At4CL2,* and *At4CL4*, participate in lignin biosynthesis, and *At4CL3* are responsible for flavonoid metabolism [7]. Moreover, five *4CL* isoforms have been characterized in rice and each isoform shows different substrate affinities in vitro [8]. Suppression of *Os4CL3* in rice leads to lignin reduction and impacts plant development and growth [8]. Similarly, downregulation of *Pv4CL1* and *Sh4CL1* reduces lignin content in switchgrass and sugarcane [9–11]. Site-directed mutagenesis of *Pp4CL1* characterizes the function of conserved amino acids, providing an insight in understanding the relationship between structure and function of 4CL proteins in *Peucedanum praeruptorum* [12].

The spontaneous *brown midrib* (*bm or bmr*) mutants in maize, sorghum, and pearl millet exhibit brownish midribs, associated with impaired lignin biosynthesis [13]. To date, at least six maize *bm* mutations (*bm1*-*bm6*) have been identified and four of them have been characterized [14–17]. The loci of maize *bm1* and *bm3* encode the cinnamyl alcohol dehydrogenase (CAD) and caffeoyl-*O*-methyltransferase (COMT), respectively, which are crucial enzymes in the lignin biosynthetic pathway [14, 17], whereas the loci of *bm2* and *bm4* are responsible for the biosynthesis of methyl donor required for the biosynthesis of both guaiacyl (G) and syringyl (S) monolignols [15, 16]. Moreover, two sorghum *bmr* loci have been identified including CAD (*bmr6*) and COMT (*bmr12* and *bmr18*) [13]. Recently, sorghum *Bmr2* has been identified to encode a major 4CL [18]. The corresponding *bm* mutant in maize, however, has yet to be found. The *bm* mutants associated with dramatic changes in lignin content and/or composition are of great interest to improve agro-industrial properties of corn stover. For example, a typical successful case was the commercial utilization of *bm3* mutant that has a high forage digestibility [13]. Thus, the characterization of novel *bm* mutations will shade light on the molecular mechanism of lignin biosynthesis, which may lead to produce more valuable sources for breeding new germplasm of forage and biofuel crops with high cell wall digestibility.

The maize *bm5* mutants had been previously identified early in 2008 [19]. The *bm5* locus was roughly mapped to chromosome 5, in the same bin as *bm1* [19, 20]. The chemical analysis further revealed that *bm5* mutation reduced the levels of Klason lignin, G monomers, and wall-bound *p*-coumarate, but increased the levels of H monomers and wall-bound ferulate in maize mature stems [21]. However, the *bm5* gene has yet to be identified, and the comprehensive effects of *bm5* mutation on lignin biosynthesis, soluble phenolics accumulation, and cell wall degradation are still elusive. In this study, we identified *Zm4CL1* as the *bm5* gene and its mutation impeded G lignin biosynthesis and, consequently, increased the level of feruloyl quinic acid (FQA) and feruloyl glycoside (FG) that are derived from ferulate, an intermediate in the lignin biosynthetic pathway in maize. Moreover, the maize mutants exhibited a normal growth and development. Finally, the disruption of lignin biosynthesis resulted in a significant increase in dry matter forage digestibility and saccharification efficiency of cell walls.

## Results

### Morphological characterization of *bm5* mutants

**Fig. 1:**
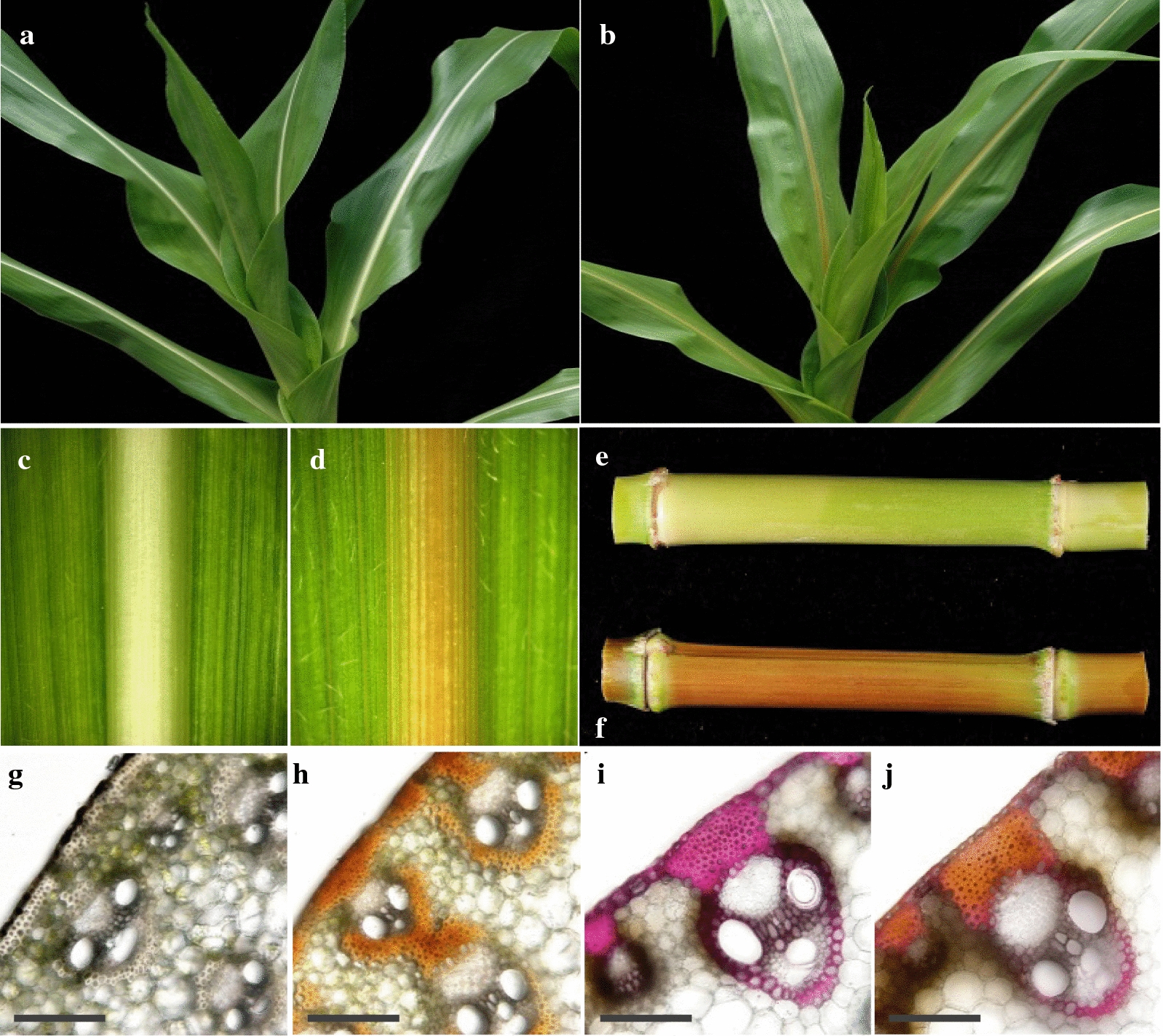
Morphological characterization of the *bm5* mutants. **a** Greenhouse grown B73 wild-type maize. **b** Greenhouse grown *bm5* mutant. **c** Adaxial view of the representative midrib of the fourth leaf collected from 60-day-old B73 wild-type maize. **d** Adaxial view of the representative midrib of the fourth leaf collected from 60-day-old *bm5* mutant. **e** The fourth internode collected from 60-day-old B73 wild-type maize. **f** The fourth internode collected from 60-day-old *bm5* mutant. **g** The midrib cross section from the fourth leaf collected from 60-day-old B73 wild-type maize without staining. Bar = 1.0 mm. **h** The midrib cross section from the fourth leaf collected from 60-day-old *bm5* mutant. Bar = 1.0 mm. **i** Phloroglucinol–HCl staining of the midrib cross section from the fourth leaf collected from 60-day-old B73 wild-type maize. Bar = 1.0 mm. **j** Phloroglucinol–HCl staining of the midrib cross section from the fourth leaf collected from 60-day-old *bm5* mutant

Three inbred stocks of *bm5* mutant were obtained from the Maize Genetic Cooperation Stock Center, designated as 504I, 504J, and 505J. The *bm5* mutants exhibited normal growth and development in our greenhouse condition except the typical reddish-brown pigmentation in the midrib and stalk compared with the wild type collected from 60 days old (Fig. 1a–f). The cross sections of midribs from *bm5* mutant and wild-type plants further revealed that the pigmentation mainly deposited in sclerenchyma tissues (Fig. 1g, h). Moreover, the above cross sections were stained with the phloroglucinol–HCl reagent. The reduced staining area of sclerenchyma tissues indicates a less lignin accumulation in the *bm5* mutant (Fig. 1i, j).

### Identification of the *bm5* gene

**Fig. 2:**
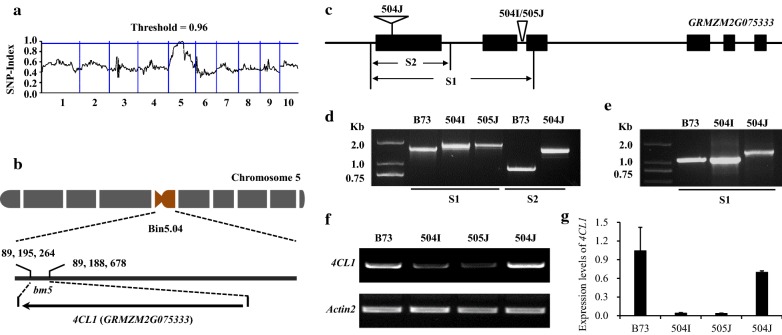
Identification of the *bm5* gene. **a** The SNP-index for the F2 progeny of *bm5*-504J. **b** Genomic location of *Zm4CL1* (*GRMZM2G075333*) on maize chromosome 5. The base pair number was referred to the Phytozome database version 1.8. **c** Exon and intron structure of the *Zm4CL1* gene. Black boxes indicate exons and lines between the boxes indicate introns. Insertion sequences of *bm5* are indicated by inverse triangles. **d** S1 PCR products amplified from B73, *bm5*-504I, and *bm5*-505J genomic DNA and S2 PCR products amplified from B73 and *bm5*-504J genomic DNA. **e** S1 PCR products amplified from B73, *bm5*-504I, and *bm5*-504J cDNA. **f** RT-PCR analysis of *Zm4CL1* expression in midribs of B73 wild-type and *bm5* mutants. **g** qRT-PCR analysis of *Zm4CL1* expression in midribs of B73 wild-type and *bm5* mutants. The primer pair was designed in 3′ untranslated region. Midribs of the fourth leaves were collected from 60-day-old B73 wild-type and *bm5* mutants

The *bm5* locus is an independent *bm* allele and has been mapped to maize chromosome 5 in the bin 5.04 region (80.8–172.4 Mb) [19, 20]. The *bm5* locus was further mapped to be linked with p-umc1591 in bin 5.04 region using simple sequence repeat (SSR) markers published in MaizeGDB database (Additional file 1: Table S1). To identify the *bm5* gene, a MutMap-assisted gene mapping based on the whole-genome sequencing technology was employed to narrow down the *bm5* locus. The maize stock *bm5*-504J was directly crossed with B73 to generate F1 individuals, and then, the F1 individual was selfed to produce F2 progeny. DNA mixture pool from about 96 F2 individuals with brown midrib phenotype was sequenced by Illumina sequencing with depth of more than 10 × coverage. By analyzing SNP-index results, the mutation region was narrowed to 80.8–120.7 Mb region on chromosome 5, and 1302 genes were in this region (Fig. 2a; Additional file 2: Table S2). Among them, only two lignin biosynthesis genes were retrieved. One is *cinnamyl alcohol dehydrogenase* (*CAD2*, *GRMZM5G844562*), the same gene as *BM1* [14]; the other candidate gene is *Zm4CL1* (*GRMZM2G075333*) encoding a 4-coumarate: coenzyme A ligase (Additional file 2: Table S2). Given that *bm1* and *bm5* were independent *bm* alleles, we suspect that *Zm4CL1* rather than *CAD2* was the most considerable *bm5* gene (Fig. 2b). To confirm our hypothesis, the full-length genomic sequences of *Zm4CL1* alleles including 5′ untranslated region, six exons, five introns, and 3′ untranslated region were obtained from *bm5* mutants and B73 by polymerase chain reaction (PCR) amplification and subsequent sequencing. Sequence alignment and PCR analysis revealed a 283 bp Mu insertion in the second intron of *Zm4CL1* in the *bm5*-504I mutant (Fig. 2c, d; Additional file 3: Fig. S1). Moreover, the same mutation of the *Zm4CL1* gene was identified in *bm5*-*505J* mutant, implying the same original *bm5* allele (Fig. 2c, d; Additional file 3: Fig. S1). Furthermore, reverse transcription polymerase chain reaction (RT-PCR) analysis showed that the Mu insertion in the second intron was properly spliced off in the transcripts during their maturation (Fig. 2e). In addition, a 658 bp Ac insertion in the first exon of *bm5*-504J allele resulted in a premature stop codon in the transcribed *Zm4CL1*, even if the mRNA was produced (Fig. 2c–e; Additional file 3: Fig. S1). Furthermore, the molecular markers designed based on the two insertion sequences were employed to determine the relationship between mutation sites and mutant phenotype (Additional file 4: Table S3). The result showed that the two independent mutation sites in *Zm4CL1* alleles were exactly associated with the same *bm* phenotype, suggesting that *Zm4CL1* is the *bm5* gene.

### Impacts of the insertions on Zm4CL1 function

**Fig. 3:**
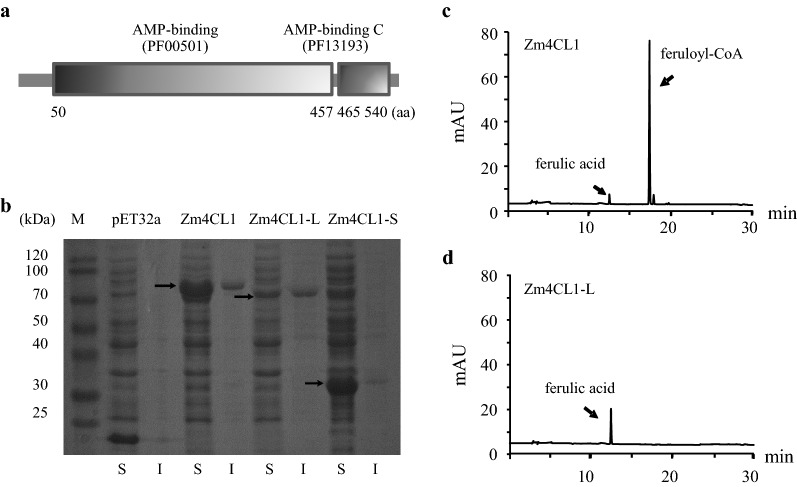
The 4CL activity of putative versions of Zm4CL1 isolated from *bm5*-504J mutant. **a** Two domains of Zm4CL1 were labeled. Image was generated using open-source SMART (http://smart.embl-heidelberg.de/). **b** SDS-PAGE analysis of recombinant Zm4CL1, Zm4CL1-L, and Zm4CL1-S proteins. S and I indicated soluble and insoluble proteins from lysate of *E. coli* cultures. The arrows showed the recombinant proteins. **c** HPLC analysis of recombinant Zm4CL1 activity against ferulate. **d** HPLC analysis of recombinant Zm4CL1-L activity against ferulate

The *bm5* near-isogenic lines (NILs) in the B73 background were generated after six backcrosses of 504I and 504J with B73, respectively. To study the impacts of the insertions on *Zm4CL1* function, we first determined the expression level of Zm4CL1 alleles in *bm5* mutants and B73. Both RT-PCR and quantitative RT-PCR (qRT-PCR) analyses revealed that the expression level of *Zm4CL1* in both *bm5*-504I and *bm5*-505J mutants was dramatically reduced compared with B73 (Fig. 2f, g). However, no difference was observed between the expression level of *Zm4CL1* in *bm5*-504J mutant and B73 (Fig. 2f, g). Furthermore, two truncated proteins were predicted and expressed in *E. coli,* since the Ac insertion caused a premature stop codon in *Zm4CL1* in *bm5*-504J mutant. The soluble protein extracts containing the truncated Zm4CL1 mutants, Zm4CL1-S (95 amino acids) and Zm4CL1-L (469 amino acids), did not exhibit a 4CL enzyme activity (Fig. 3, Additional file 5: Fig. S2).

**Fig. 4:**
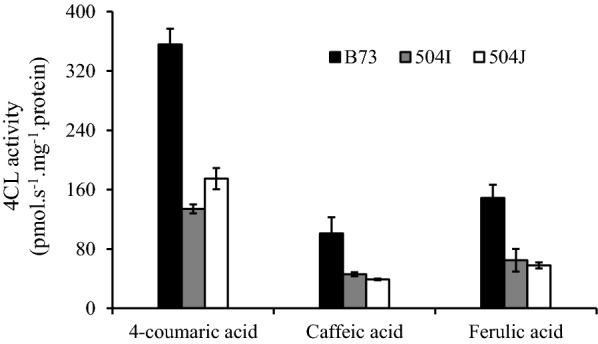
Effect of Zm4CL1 mutations on 4CL enzyme activities. Crude enzyme extracts were prepared from the midribs of B73, *bm5*-504I, and *bm5*-504J. The 4CL enzyme activities of the above crude enzyme extracts against *p*-coumarate, caffeate, and ferulate were determined. Midribs of the fourth leaves were collected from 60-day-old B73 wild-type and *bm5* mutants

Furthermore, the soluble crude plant proteins were extracted from the midribs of *bm5*-NIL mutants and B73 wild-type plants. The activities of extracted proteins were analyzed using *p*-coumarate, caffeate, and ferulate substrates. The results revealed a dramatic reduction in 4CL enzyme activities of the extractable crude proteins from *bm5* mutants compared with B73 wild-type plants (Fig. 4). The 4CL activities for all the used substrates were reduced by 51–62% in *bm5*-504I and *bm5*-504J mutants (Fig. 4).

### Phylogenetic and expression pattern analysis of *Zm4CL* genes

The *4CL* family consists of a cluster of small and conserved genes in plants, playing a crucial role for phenylpropanoid biosynthesis. Five *Zm4CLs* were retrieved in maize genome, and a phylogenetic tree was built using 4CL deduced amino acid sequences from six species. 4CLs were clustered into two main groups, groups I and II, and the members from the dicots and monocots were gathered separately in each subgroup (Additional file 6: Fig. S3). Zm4CL1 belongs to group I and is closely clustered with rice Os4CL3 (LOC_Os02g08100) and sorghum BMR2 (Sb04g005210), implying a conserved function in lignin biosynthesis (Additional file 6: Fig. S3). Furthermore, the RNA sequencing data were downloaded from the MaizeGDB database and analyzed, which revealed a higher expression level of *Zm4CL1* in all tested vegetative tissues compared with the other *Zm4CL* genes, particularly in well-lignified root and internode tissues (Additional file 7: Table S4).

### Effects of *Zm4CL1* mutation on lignin biosynthesis

We assessed the global effects of *Zm4CL1* mutation on lignin biosynthesis by Affymetrix microarray analysis. Compared with B73 wild-type plant, a total of 57 transcripts, many of which are involved in the process of carbohydrate metabolism, cellular metabolites, primary metabolites, and macromolecule metabolites, were differentially expressed in *bm5*-504J mutant (Additional file 8: Fig. S4, Additional file 9: Table S5). However, none of genes were involved in monolignol biosynthesis, implying that the disruption of *Zm4CL1* did not trigger the expression of other genes related to lignin biosynthesis in the mutant except its paralogs (Additional file 10: Table S6). The expression level of *Zm4CL1* paralog, *GRMZM2G174732*, was increased in both *bm5*-504I and *bm5*-504J mutants. In contrast, the expression levels of other *Zm4CL1* paralogs including *GRMZM2G054013*, *GRMZM2G048522*, and *GRMZM2G055320* were only increased in the *bm5*-504J mutant, but did not exhibit a similar change in the *bm5*-504I mutant (Additional file 11: Fig. S5).

We next examined lignin content and composition to investigate the effects of *Zm4CL1* mutation on lignin biosynthesis. Extractive free cell wall residues (CWRs) were employed to analyze the total lignin content and composition. Our results revealed a similar amount of total lignin between *bm5*-504J mutant and B73 wild-type plant as measured by the acetyl bromide (AcBr) method (Additional file 12: Table S7). Lignin composition analysis further showed that G lignin was dramatically reduced in *bm5*-504J, which only amounted to 42.3% of the control (Additional file 12: Table S7). The content of S lignin, however, has no significant change, as a consequence, the S/G ratio raised dramatically from 0.58 in B73 to 1.47 in *bm5*-504J (Additional file 12: Table S7). In contrast, the yield of H lignin was increased in the mutant (Additional file 12: Table S7). In addition, the relative percentage of H and S lignins were remarkably increased from 4.3% (H/H + G+S) and 35.1% (S/H + G+S) in B73 to 7.8% and 54.9% in the mutant, whereas G lignin still remained at a low level (37.3% in *bm5* versus 60.5% in B73) (Additional file 12: Table S7).

### Soluble phenolic characterization of *bm5* mutants

**Fig. 5:**
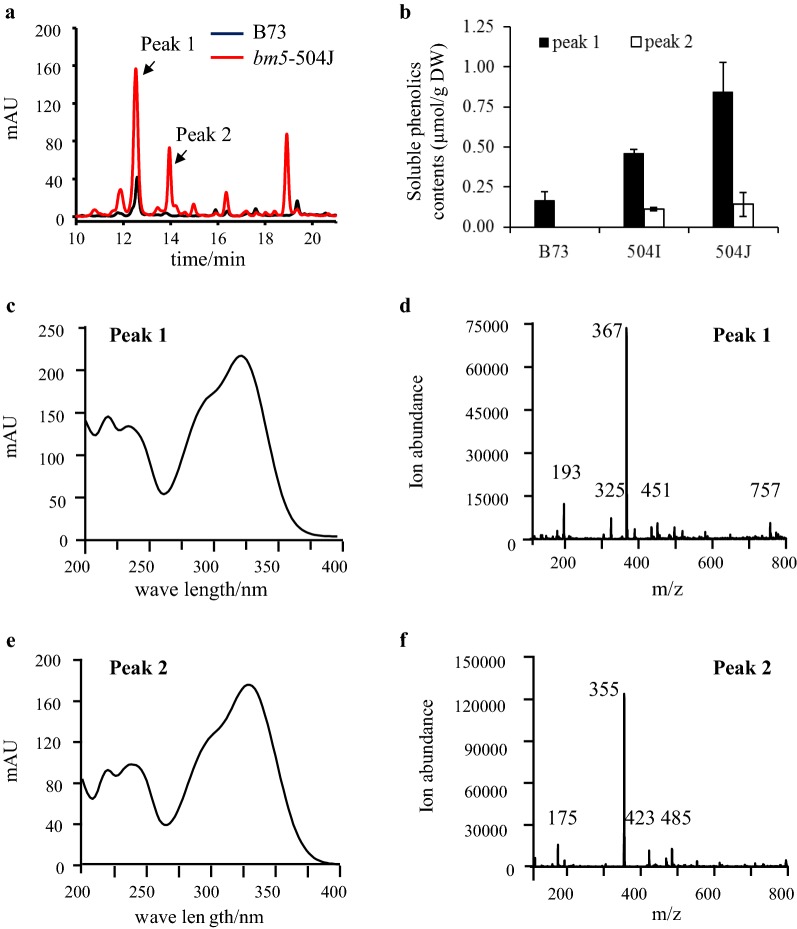
Effects of Zm4CL1 mutations on phenylpropanoid accumulation in *bm5* mutants. **a** The profile of soluble phenolics in methanolic extracts from midribs of *bm5*-504J mutant and B73 wild-type maize. The profile of soluble phenolics was performed by LC–PDA–ESI-MS/MS. **b** The contents of FQA (peak 1) and FG (peak 2) in *bm5* mutants and B73 wild-type maize. Midribs of the second-to-fifth leaves from the top were collected from 60-day-old B73 wild-type maize, *bm5*-504I and *bm5*-504J mutants. Values are mean ± SE (*n* = 3). DW, dry weight. FQA, feruloyl quinic acid. FG, feruloyl glucoside. **c**–**f** The remarkably accumulated phenolics in the *bm5* mutant were preliminarily identified as FQA (peak 1) and FG (peak 2) based on their UV–visible spectra (**c** and **e**) and mass spectra (**d** and **f**)

**Fig. 6:**
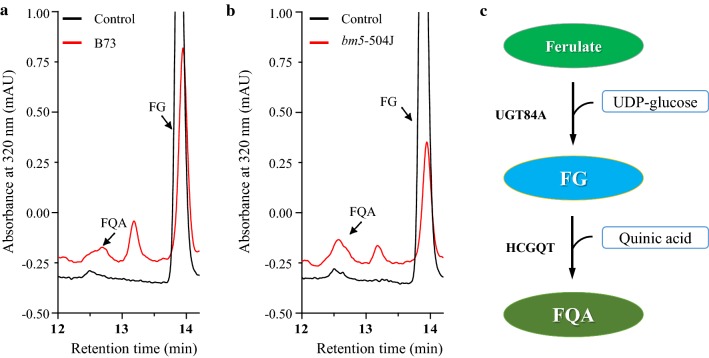
The putative biosynthetic pathway of FQA in maize. **a** The formation of FQA after adding FG into the crude extractive protein from B73 plant. **b** The formation of FQA after adding FG into the crude extractive protein from *bm5*-504J mutant. **c** The putative FQA biosynthetic pathway in maize. FQA, feruloyl quinic acid. FG, feruloyl glucoside. HCGQT, hydroxycinnamoyl d-glucose: quinate hydroxycinnamoyl transferase

To gain the effects of lignin disruption on its intermediates, the soluble phenolics were extracted from midribs and characterized by soluble phenylpropanoid profiling through reversed-phase liquid chromatography coupled with photo-diode array detection and electrospray ionization tandem mass spectrometry (LC–PDA–ESI-MS/MS). An approximate 2.7–5.0-fold increase in FQA (peak 1) was revealed in *bm5*-504I and *bm5*-504J mutants (Fig. 5a–d). Most importantly, we identified one novel metabolite (peak 2) present in the mutant, but absent in B73 (Fig. 5a). The analysis of UV–visible and mass spectra further showed that the peak 2 was the glucoside derivative of ferulate (Fig. 5e, f, Additional file 13: Table S8). Furthermore, the compound of peak 2 was confirmed to be FG by comparing with the standard FG produced by AtUGT84A1 toward ferulate and UDP-glucose using LC–PDA–ESI–MS/MS (Additional file 13: Table S8, Additional file 14: Fig. S6, Additional file 15: Fig. S7b, Additional file 16: Fig. S8a–c). Moreover, the *AtUGT84A1* homologous genes, *ZmUGT84A*-*1* (GRMZM2G417945) and *ZmUGT84A*-*2* (GRMZM2G304712), were isolated from maize and expressed in *E. coli*. As expected, the soluble protein extracts containing ZmUGT84A-1 and ZmUGT84A-2 converted ferulate and UDP-glucose into FG in vitro (Additional files 15: Fig. S7c, d, 16: Fig. S8a–c). To gain an insight on the possible biosynthetic pathway of FQA in maize, we added FG into the crude extractive protein of maize midribs from *bm5*-504J mutant and B73 wild-type plant. As expected, the crude extractive proteins from *bm5*-504J mutant and B73 plant had capacity to catalyze the formation of FQA (Fig. 6, Additional file 16: Fig. S8d, f, Additional file 17: Fig. S9).

### Disruption of Zm4CL1 improved cell wall digestibility

**Fig. 7:**
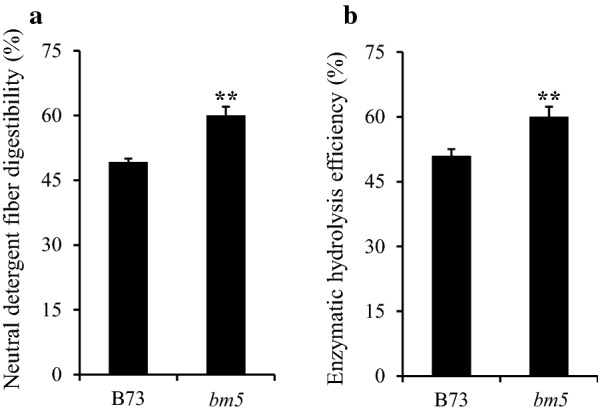
Effects of *bm5* mutation on cell wall digestibility. **a** Forage digestibility of cell walls of *bm5* mutant and B73 wild-type maize. **b** Saccharification efficiency of cell walls of *bm5* mutant and B73 wild-type maize. Stalk samples were collected from 90-day-old *bm5*-504J mutant and B73 wild-type plants. Values are mean ± SE (*n* = 3). One or two asterisks indicate significance corresponding to *p* < 0.05 or 0.01 (Student’s *t* test)

Given that lignin is a crucial factor negatively impacting lignocellulosic biomass utilization, we evaluated the effects of the altered lignin biosynthesis resulted from the *bm5* mutation by a fast and nondestructive near infrared reflectance spectroscopy (NIRS) method. Disruption of *Zm4CL1* led to a significant reduction in acid detergent lignin (ADL), whereas it had no effects on cellulose and hemicellulose accumulation (Additional file 18: Table S9). To study if the dramatically altered lignin biosynthesis could improve forage digestibility of the *bm5* mutants, neutral detergent fiber digestibility (NDFD) frequently employed for evaluating or marketing forages was evaluated. Our result revealed a substantially improved forage digestibility (i.e., a relative increase of 22.0%) in the mutants compared with B73 wild-type plants (Fig. 7a). In addition, we also studied the effect of lignin alteration on the degradation efficiency of cell wall polysaccharides. Our result showed that the saccharification efficiency was increased from 51.0% in B73 to 60.0% in the *bm5* mutant (i.e., a relative increase of 17.6%) (Fig. 7b).

## Discussion

The spontaneous *bm5* mutation was identified in 2008 and roughly mapped to maize chromosome 5 [19, 21, 22]. In our work, the *bm5* mutant gene *Zm4CL1* was identified to encode a 4-coumarate: coenzyme A ligase through a MutMap-assisted gene mapping based on the whole-genome sequencing technology. Mutation of *Zm4CL1* resulted in a typical brown midrib phenotype accompanied by the alteration of lignin and soluble phenolics in *bm5* mutants. Moreover, the disruption of lignin biosynthesis led to a significant improvement in both forage digestibility and saccharification efficiency of cell wall polysaccharides. Thus, our results suggest that *Zm4CL1* is a potential target for cell wall engineering, and the *bm5* mutants are interest sources that could be integrated into breeding for development of novel germplasm with a high cell wall conversion rate.

The expression level of *Zm4CL1* was relatively higher than the other four *4CL* genes, especially in the well-lignified tissues of B73 wild-type plants. The phylogenetic tree analysis shows that Zm4CL1 is clustered with BMR2, Os4CL3, and Pv4CL1, which are the major genes responsible for lignin biosynthesis of sorghum, rice, and switchgrass [8, 11, 18]. Taken together, our results imply that *Zm4CL1* was a major isoform involved in lignin biosynthesis in maize. Moreover, two types of insertion mutations were found in the three *bm5* mutants. One is Mu transposon insertion in the second intron of *Zm4CL1* of *bm5*-504I and *bm5*-505J, which had potential to reduce the expression levels of *Zm4CL1* dramatically and, consequently, reduced the 4CL activity in maize. The previous studies have suggested that introns can significantly regulate gene expression in many direct and indirect ways in plants [23]. It is not surprising that the Mu insertion in the intron of *Zm4CL1* of *bm5*-504I and *bm5*-505J might have a negative influence on the gene expression and/or trigger a rapid mRNA decay in maize. The function of introns in *Zm4CL1* is currently unclear, but it deserves further investigation. The other is Ac transposon insertion in the first exon of *Zm4CL1* of *bm5*-504J, which caused a premature stop codon in the transcribed *Zm4CL1*. The shortage of 4CL activities was observed in crude protein extracts from midribs of the *bm5*-504J mutant, suggesting that the Ac transposon insertion can significantly reduce the activity of Zm4CL1 in maize. In addition, the insertion of Ac transposon at the first exon of *Zm4CL1* may produce two truncated proteins as compared with a normal 555 aa Zm4CL1. The shorter version (Zm4CL1-S) remains only the N terminal 95 aa; The longer one (*Zm4CL1*-*L*) could be translated from the first ATG following the insertion sequence and produces a 469 aa protein lacking the partial AMP-binding domain at the N terminal. We further expressed the two nucleic acid sequences in *E. coli.* The enzyme activity assay of the soluble protein extracts containing the truncated Zm4CL1 mutants showed that the two truncated versions lost the 4CL function totally. These results demonstrate that the Ac transposon insertion at the first exon accounted for the reduction of 4CL activity in the crude protein extracted from *bm5*-504J mutant.

The impact of *bm5* mutation on maize lignin had been investigated by Méchin et al. (2014) [21]. Consistent with the previous study, our lignin analysis data suggest that the biosynthesis of G lignin was dramatically impaired in the *bm5* mutants, whereas the H lignin was significantly increased. Although the content of S lignin was little affected in *bm5* mutant, the relative percentage of S lignin was still higher than that of B73 wild type. The previous study also revealed a significant reduction in Klason lignin content [21]. However, no difference between *bm5* mutant and B73 wild type was observed in our work. One possible explanation for the observed effect on the total lignin content is that we used midribs rather than mature stems for lignin analysis besides different methods employed for the measurement of total lignin content. In addition, the content of H lignin was significantly increased in *bm5* mutants, which could partially compensate for the reduction of G lignin. The previous study has suggested that at least four *4CL* paralogous exist in sorghum genome, and the expression levels of these *4CLs* including *BMR2* are elevated in sorghum *Bmr2* mutants [18]. Our results also showed that the expression of *Zm4CL2* (*GRMZM2G174732*) were increased two-to-four-fold in the two *bm5* mutants. In particularly, the loss function of *bm5*-504J mutation raised the expression levels of the other *Zm4CLs* significantly. Therefore, we suspected that other 4CLs could compensate for the shortage of 4CL activity against *p*-coumarate in *bm5* mutants, leading to an increase in H lignin content, since *p*-coumarate rather than caffeate and ferulate is the preferred substrate of the 4CLs [18].

Monolignols are synthesized through a complexity metabolic grid linked to the phenylpropanoid biosynthetic pathway in plants. Therefore, the disruption of lignin biosynthesis has a possibility to shunt its intermediates towards some important soluble phenolic metabolites in plants. A 2.7–5.0-fold increase in FQA was found in the *bm5* mutants. FQA is a valuable antioxidant compound for plants, animals, and human. The previous studies have suggested that feruloyl-CoA is the activated intermediate in *Solanaceae* species, whereas FG is the activated one in other species [24]. Abundant FQA accumulates in maize, which can accumulate in the thrip-resistant cultivars of tomato or be induced in infected maize [25, 26]. The biosynthetic pathway of FQA, however, still remains largely elusive in this species. FG was a novel phenolic compound present in the *bm5* mutants, implying that a biosynthetic pathway exists in maize through FG towards FQA. Moreover, we isolated two UDP-glucoside transferase genes, *ZmUGT84A*-*1* and *ZmUGT84A*-*2*, which were involved in the glycosylation of ferulate in vitro (Additional file 11: Fig. S5c, d). Taken together, our results suggest that the *bm5* mutation can redirect the redundant ferulate towards FQA through FG biosynthetic pathway.

Disruption of *Zm4CL1* significant reduced ADL and, therefore, improves forage digestibility in the *bm5* mutant. Consistently, cell wall enzymatic hydrolysis assay indicates that the mutation of *Zm4CL1* can increase saccharification efficiency of cell wall polysaccharides significantly as well. Although *Zm4CL1* mutation impaired lignin biosynthesis, the AcBr lignin content was not changed. It is not surprising, because the AcBr method reveals more lignin and its derivatives than the ADL procedure [27]. In addition, the alteration of lignin composition did not affect the contents of cellulose and hemicellulose, suggesting a potential value of the *bm5* mutants for lignocellulosic biomass utilization.

## Conclusion

The spontaneous *bm* mutations can provide useful resources for commercial utilization of corn stover. We characterized *Zm4CL1* as the mutation gene of maize *bm5*. Disruption of *Zm4CL1* impaired lignin biosynthesis in *bm5* mutants and improved the cell wall hydrolysis efficiency. Like *bm3*, the *bm5* mutants will probably to be of interest to be candidates for breeders in the future.

## Methods

### Plant materials and growth conditions

The maize stocks, 504I (*bm5*-*PI251930*), 504J (*bm5*-*PI262480*), and 505J (*bm5*- *PI251893*) containing the *bm5* alleles, were obtained from the Maize Genetics COOP Stock Center. The *bm5* NILs developed following a six backcrosses of 504I, 504J, and 505J with B73, respectively, were used for characterization of *bm5* gene. The *bm5*-504I and *bm5*-504J NILs were employed for further phenotypic, molecular, and biochemical analysis. The maize plants were grown in the greenhouse at 26 °C with 16 h light (390 µE m^−2^ S ^−1^).

### Genetic analysis and MutMap-assisted gene mapping

Genomic DNA was extracted from *bm5*-504J by the 2× CTAB method [28]. To confirm the mutant loci, 12 available SSR markers designed in bin 5.04 region were randomly selected and downloaded from MaizeGDB database (Additional file 1: Table S1). Among them, one SSR marker (p-umc1591) was linked with the *bm5* phenotype and used to confirm the previous mapping region and identify the homozygous progeny (Additional file 1: Table S1).

The homozygous progeny from *bm5*-504J stock was directly crossed with B73 to generate F1, followed by selfing F1 individuals to generate F2 progeny. DNAs from 96 F2 individuals with the typical brownish midrib phenotype were pooled in an equal ratio and sequenced by Illumina sequencing with depth of more than 10 × coverage. The short reads were then aligned to the reference B73 genome sequences. The single-nucleotide polymorphism markers were analyzed and the SNP-index for each SNP was calculated to quantify the nucleotides difference from the reference B73 genome sequence. The genomic region with SNP-index = 1 corresponds to the potential mutant position of the *bm5*.

### Cloning and expression analysis of *Zm4CL1*

*Zm4CL1* genomic sequences from *bm5* mutants (504I, 504J, and 505J) and B73 wild-type plants were isolated and sequenced (Additional file 4: Table S3). The primer pairs S1-F + R and S2-F + R were designed for rapid detection of the insertion sequences in the mutants (Fig. 2c, Additional file 4: Table S3). Midribs of the second-to-fifth leaves from the top were collected from 60-d old *bm5* mutants and B73 wild-type plants. Total RNAs were extracted from the above midribs, and expression levels of *Zm4CL1* were quantified by RT-PCR and qRT-PCR as described by Tang et al. (2014) [16]. The primer pairs for RT-PCR and qRT-PCR were designed around the 3′ untranslated region of *Zm4CL1* (Additional file 4: Table S3). To study the influence of the insertions on mature mRNAs, the cDNA fragments of *Zm4CL1* were amplified by RT-PCR from *bm5*-504I and *bm*5-504J mutants with the primer pair S1-F + R (Additional file 4: Table S3). To determine the expression levels of other *Zm4CLs*, the qRT-PCR primer pairs were designed in their 3′ untranslated region (Additional file 4: Table S3).

### Phylogenetic analysis of 4CLs

All 4CL protein sequences were downloaded from Phytozome database (http://www.phytozome.net), and the alignments were carried out using CLUSTAL_X [29]. The phylogenetic tree was built using the neighbor-joining method in MEGA 5.0 [30]. Bootstrap values were calculated with 1000 iterations and the values under 70% were cut off.

## 4CL enzyme activity assay of maize crude extractive protein

Midribs of the second-to-fifth leaves from the top were collected from 60-day-old *bm5* mutants and B73 wild-type plants. Powdered fresh midribs (~ 500 mg) were extracted for 3 h at 4 °C in protein extraction buffer [31]. The samples were centrifuged at 17,900×*g* for 20 min at 4 °C, and the extracts were desalted on PD-10 columns (Pharmacia) and used for 4CL enzyme activity assay against *p*-coumarate, caffeate, and ferulate as described by Liu et al. [31].

### Enzyme activity assay of soluble protein extracts of Zm4CL1 proteins

The coding region of *Zm4CL1* was amplified from cDNAs of *bm5* mutants and B73 wild-type plants using the primers in Additional file 4: Table S3. *Zm4CL1*-504J would be translated in two versions, Zm4CL1-S and Zm4CL1-L, resulted from the Ac transposon insertion in the first exon. The PCR products of *Zm4CL1*-*S*, *Zm4CL1*-*L*, and *Zm4CL1* were subcloned into the pET32a vector (Additional file 4: Table S3). The constructs were introduced into *Rosetta E. coli* cells for recombinant protein expression [32]. The 4CL enzyme activities against *p*-coumarate, caffeate, and ferulate were determined as describe by Liu et al. [31].

### Microarray analysis

The midribs were separated from the leaves of 60-day-old *bm5*-504J mutants and B73 wild-type plants. RNA extraction and purification, probe labeling, hybridization, and scanning for Affymetrix microarray analysis were conducted as previously described [4].

### Lignin content and composition analysis

Midribs of the fourth-to-fifth leaves from the top were collected from 60-day-old *bm5* mutants and B73 wild-type plants. Soluble extracts were removed from the ground lyophilized samples by four successive extractions with chloroform/methanol (2:1 v/v), methanol, methanol/H_2_O (1:1 v/v), and water at room temperature as described by Chen and Dixon [1], and the remaining CWRs were lyophilized for lignin analysis. The quantification of lignin content was conducted by acetyl bromide method [33]. The lignin compositions were measured by thioacidolysis method [34, 35].

### Profiling analysis of soluble phenolics

Midribs of the second-to-fifth leaves from the top were collected from 60-day-old *bm2*-*ref* mutants and B73 wild-type plants and homogenized in liquid nitrogen and lyophilized. The methanolic extracts from lyophilized materials including B73 wild type, *bm5*-504I and *bm5*-504J mutants, were subjected to soluble phenolic profiling analysis by LC–PDA–ESI-MS/MS [36]. The phenolic compounds were identified based on their UV–visible spectra, mass spectra, and comparison with the authentic standard compound and the MS data reported by Eloy et al. [37]. The authentic standard compounds, *p*-coumarate, caffeate, and ferulate, were ordered from Sigma-Aldrich (St. Louis, MO, USA). The FG was prepared using the production of the identified AtUGT84A1 toward ferulate and UDP-glucose [38]. Moreover, the AtUGT84A1 homologous genes, ZmUGT84A-1 and ZmUGT84A-2 isolated from B73 wild type, were cloned into pET32a vector, respectively, to produce recombinant proteins in *E. coli* [32]. Primers for gene cloning and vector construction were listed in the Additional file 4: Table S3. The soluble protein extracts containing ZmUGT84A-1 and ZmUGT84A-2 identified by SDS-PAGE (Additional file 14: Fig. S6) were used to convert ferulate and UDP-glucose into FG in vitro [37]. The purified FG through HPLC was further identified by LC–PDA–MS/MS [36]. In addition, FQA was synthesized from FG and quinic acid after incubation with the crude extractive proteins from midribs of *bm5* mutant as described by Villegas and Kojima (1986) [39].

### Cell wall digestibility analysis

Stalk samples were collected from *bm5*-504J mutants and B73 wild-type plants at the R1 stage (silk emergence) and dried in an oven at 40 °C for 1 week. Samples were ground through a Wiley mill with a 1-mm sieve for analysis of neutral detergent fiber (NDF), acid detergent fiber (ADF), and in vitro true dry matter digestibility (IVTDMD) using NIRS [40]. ADL was measured using an ANKOM 200 Fiber Analyzer (ANKOM Technology Corp.) [41]. ADL, NDF, and ADF were used to calculate cellulose (ADF-ADL) and hemicellulose (NDF–ADF) contents. IVNDF, ADF, and IVTDMD were employed for IVNDFD calculation by the formula (NDF + IVTDMD-100)/NDF × 100.

Saccharification of maize stalk samples was performed following the analytical procedure described by the National Renewable Energy Laboratory (LAP-009: Enzymatic Saccharification of Lignocellulosic Biomass). Briefly, solubilized sugars were yielded from CWRs digested by pretreatment with 1.5% H_2_SO_4_ at 121 °C for 40 min and then exposure to a cellulase and cellobiase mixture for 72 h after washing with Milli-Q water. The solubilized sugars were detected with the phenol–sulfuric acid assay method [42].

### Statistical analysis

Samples were collected from three biological replicates. The mean values were used for statistical analyses. Data from each trait were subjected to Student’s *t* test. The significance of treatments was tested at the *p* = 0.05 and 0.01 levels. Standard errors were provided in all tables and figures as appropriate.

## Additional files

**Additional file 1: Table S1.** The SSR markers used in this study.

**Additional file 2: Table S2.** SNPs with SNP-index 1 within the candidate genomic region detected on chromosome 5.

**Additional file 3: Fig. S1.** Insertion sequences of the *Zm4CL1* in *bm5* mutants.

**Additional file 4: Table S3.** Primers used in this study.

**Additional file 5: Fig. S2.** The 4CL activity of soluble protein extracts containing Zm4CL1 and the truncated Zm4CL1 mutants.

**Additional file 6: Fig. S3.** Phylogenetic analysis of 4CLs in vascular plants.

**Additional file 7: Table S4.** Expression levels of *Zm4CL* genes in maize different tissues.

**Additional file 8: Fig. S4.** Transcriptome analysis of *bm5* mutant by microarray.

**Additional file 9: Table S5.** Signal intension of the probe sets of regulated genes in *bm5*-504J mutant and B73 wild-type plant.

**Additional file 10: Table S6.** Signal intension of the probe sets of lignin genes in *bm5*-504J mutants and B73 wild-type plants.

**Additional file 11: Fig. S5.** Expression levels of other *4CL* paralogs in *bm5* mutants.

**Additional file 12: Table S7.** Lignin content and composition of *bm5* mutant.

**Additional file 13: Table S8.** LC–PDA–ESI-MS/MS identification of soluble phenolics in methanolic extracts from midribs of the *bm5* mutant.

**Additional file 14: Fig. S6.** SDS-PAGE analysis of recombinant AtUGT84A1, ZmUGT84A-1, and UGT84A-2 proteins.

**Additional file 15: Fig. S7.** The characterization of UDP-glucoside transferase forming glucose ester with ferulate in vitro.

**Additional file 16: Fig. S8.** The biosynthesis and characterization of FG and FQA in vitro.

**Additional file 17: Fig. S9.** UV–visible spectra of the remarkably accumulated products formed after adding FG into the crude extractive proteins from B73 and *bm5*-504J mutant.

**Additional file 18: Table S9.** ADL, hemicellulose, and cellulose contents of *bm5* mutants and B73 wild-type plants.

## Abbreviations

AcBr: acetyl bromide
ADF: acid detergent fiber
ADL: acid detergent lignin
*bm*: brown midrib
CAD: cinnamyl alcohol dehydrogenase
4CL: 4-coumarate: CoA ligase
COMT: caffeic acid *O*-methyltransferase
CWR: cell wall residue
FG: feruloyl glycoside
FQA: feruloyl quinic acid (FQA)
G: guaiacyl
IVTDMD: in vitro true dry matter digestibility
LC–PDA–ESI-MS: liquid chromatography coupled with photo-diode array detection and electrospray ionization tandem mass spectrometry
NDF: neutral detergent fiber
NDFD: neutral detergent fiber digestibility
NIL: near-isogenic lines
NIRS: near infrared reflectance spectroscopy
PCR: polymerase chain reaction
RT-PCR: reverse transcription polymerase chain reaction
qRT-PCR: quantitative transcription polymerase chain reaction
S: syringyl
SSR: simple sequence repeat
