# Supplementary material for: Mutation of 4-coumarate: coenzyme A ligase 1 gene affects lignin biosynthesis and increases the cell wall digestibility in maize brown midrib5 mutants
Source: Biotechnol Biofuels. 2019 Apr 10;12:82. doi: 10.1186/s13068-019-1421-z (PMC6456989; doi:10.1186/s13068-019-1421-z)
Supplement: Supplementary file 1 — Additional file 1: Table S1. The SSR markers used in this study. [file 13068_2019_1421_MOESM1_ESM.docx]

**Additional file 1: Table S1.** The SSR markers used in this study.

| SSR markers | Forward primer sequences (5'-3') | Reverse primer sequences (5'-3') |
| --- | --- | --- |
| p-umc2298 | ATCCACTCCCAAGTCCCAACAC | CTTCTTCCGGTTCTTCTTCAGGC |
| p-umc1110 | TTACACCAAGGTCCGAAACAAGAT | TCTTGGAAGGCAAGACTCTACCTG |
| p-umc1591 | CAACCAACTGGCAACTACTCGAC | GAGGTCTCTCTCGGTCGACATC |
| p-umc1575 | GCCTAGACGTCATGGACAACG | GAGTCGAGACTGCCGTCCTTC |
| p-umc1224 | CTGAGAGGTCCCAAAGGAGTACAA | ATGACCTGCACACAGAAAGAACAA |
| p-umc2300 | ACAAGTTAACAGAACCATACGGGG | TTTGTCTTGAGTGCCAATTTGAGA |
| p-umc2373 | ACCCAAGTGAGGTGAAGTGAAGC | TATGGTACAGGCACAGCAGCAGTA |
| p-umc1815 | ACATACAGGTCACAACTCACAGCG | GCTGCCTTCTTCCTTCTCTTCTCT |
| p-umc2301 | AATGCGTTGTGCTGTGAAATG | CTAGAAGCTACAGCGAGTGAGGAC |
| p-umc1624 | GAGAGGTCGTCGTCGCTACTG | GAGACCAGATTCTTGGAACGGTAA |
| p-umc2299 | CAGCAACTACACGAGTCAACACAG | GAATTTGTTGGAGGAATCGAAGAG |
| p-bnlg653 | CGCATTGCCATGGATGAAGAACTGG | GCAAGCGCCTCACAAGGTATGCACA |
