# Supplementary material for: Mutation of 4-coumarate: coenzyme A ligase 1 gene affects lignin biosynthesis and increases the cell wall digestibility in maize brown midrib5 mutants
Source: Biotechnol Biofuels. 2019 Apr 10;12:82. doi: 10.1186/s13068-019-1421-z (PMC6456989; doi:10.1186/s13068-019-1421-z)
Supplement: Supplementary file 3 — Additional file 3: Fig. S1. Insertion sequences of the Zm4CL1 in bm5 mutants. [file 13068_2019_1421_MOESM3_ESM.docx]

**Additional file 3: Fig. S1** Insertion sequences of the *Zm4CL1* in *bm5* mutants

**a**

**b**

ACGCGTTCCCCAACTTTTTTGCATTTTAGCCCTTTTAGGATTTCTTTTGCACAATTATGCCCTTTGTAGTTTCATTTCAAAAAATGGACCCTTACCTCGGCGCCGTCATCATTGGCGCCGAGGTAACACATAGTGGCGCCAATGGTACTGGCGTCGATGTGTGTTACCTCGGCGCCAATGATGATGGCGCCGAGGTTGGGGTCCATTTTTTAAAATGAAACTGTAAAGGGCATAGTTGTGCAAAAAAAAACTCAAAAAAGGCTAAAATTCAAAAAAGTTGGCACGCGTTC

**c**

**d**

CCTGTCCCTAGAGATGTCCAAACGGGCCGGCCCGGTAGTGGGCCCGTGACCCGGCACGGTCGTGGCCCGGCACGAGCACGGCACGGCCCGACTAGAGGCGTGCCCGGGCCGGCCCGGTTCAATAGCCGTGCCGGGCTTGGGCTAGCCGTCGGGCCCGCGGTGCAGGCACGGGCCCGACACGGTTAATTGGTAGGCACGGTAACGGCCCGTTTGAGGGACTGATTTTATAACGGTCAGTCTATTTTTAGCTATAATACTTATGATATGTTATTATATGAGAGCATTCTGACTTGTAATATGTGTTTATATGTGTATCTAAAATTCTTTTGTCTAAATATGTATCTAAAATTATAAATATAATTAATTAAATCTAAAATAAATTTGAATATAACATTTAAATTCTGAATTTTGAAGTGTTTTTTTTGTTTATGGGACCGGGCCCGACCCGACACGACAGGCCCGCCGTGCCTCCGGCCCGGCACGGCCCGTGTAAGAATAACCGTGCCGGGCTTGGGCCGCGTTAGCCGGCCCACGGGCCGGCCCGGCACGGCACGATACCAAACCGTGCCGGGCTTGGACTAGTGCCTCTCGTGCCGGGCCAAGCCGTGCCCGGGCCGGGCCGGCCCGGCACGGCCCATTGGACATGTATACCTGTCCC

**Additional file 3: Fig. S1** Insertion sequences of the *Zm4CL1* in *bm5* mutants.

**a.** Sequence alignment between partial Zm4CL1 sequences of B73, *bm5*-504I, and *bm5*-505J. **b.** Mu transposon sequences inserted in the second intron of *Zm4CL1* in *bm5*-504I and *bm5*-505J mutants. The target repeats and inverted repeats of the transposable element are highlighted in gray and yellow color, respectively. **c.** Sequence alignment between partial Zm4CL1 sequences of B73 and *bm5*-504J. **d.** Ac transposon sequences inserted in the first intron of *Zm4CL1* in *bm5*-504J mutant. The transposable element was highlighted in yellow color. The stop codon in the insertion sequence was represented with black rectangular box.
