# Supplementary material for: Mutation of 4-coumarate: coenzyme A ligase 1 gene affects lignin biosynthesis and increases the cell wall digestibility in maize brown midrib5 mutants
Source: Biotechnol Biofuels. 2019 Apr 10;12:82. doi: 10.1186/s13068-019-1421-z (PMC6456989; doi:10.1186/s13068-019-1421-z)
Supplement: Supplementary file 4 — Additional file 4: Table S3. Primers used in this study. [file 13068_2019_1421_MOESM4_ESM.docx]

**Additional file 4: Table S3.** Primers used in this study.

| Name | Forward primer sequences (5'-3') | Reverse primer sequences (5'-3') |
| --- | --- | --- |
| Zm4CL1-S1 | AAGGTCTGAGATGGGTTCCG | CCTGCCTCCGTCATCCCGT |
| Zm4CL1-S2 | AAGGTCTGAGATGGGTTCCG | CACGCCATTATTTATGAGCC |
| Zm4CL1-ORF | ATCCGACAAGCCAGTCCA | CCGTAATCAGGCTTGTTCTTT |
| Zm4CL1-RT | TACGGGATGACGGAGGCAG | CGTAATCAGGCTTGTTCTTTGG |
| Zm4CL1-3'UTR-qRT | TCTTCACCGAATCCATCCCCA | CAGGAATACGTGTCCCTTCTGTTG |
| ZmUbiq-qRT | GTTTAAGCTGCCGATGTGCCTG | GACACGACTCATGACACGAACAGC |
| Zm4CL1-32a | GATATCGGATCCGAATTCATGGGTTCCGTAGACG | CGACGGAGCTCGAATTCTCAGTGAACACCGGCGGCGA |
| Zm4cl1-l-32a | GATATCGGATCCGAATTCATGGGGGTGGGCAAGG | CGACGGAGCTCGAATTCTCAGTGAACACCGGCGGCGA |
| Zm4cl1-s-32a | GATATCGGATCCGAATTCATGGGTTCCGTAGACG | CGACGGAGCTCGAATTCTCACGGGCCCACTACCGG |
| ZmUGT84A-1-32a | gctGATATCGGATCCGAATTCATGGGAGAGGAAGAGGGC | ttgtCGACGGAGCTCGAATTCAACTAGGACTAGGAATC |
| ZmUGT84A-2-32a | gctGATATCGGATCCGAATTCATGGGAGAGGAAGCGGCC | ttgtCGACGGAGCTCGAATTCAGGTTTGGACTCTGACC |
| AtUGT84A1-32a | gctGATATCGGATCCGAATTCATGGGATCCATATCAGAAA | ttgtCGACGGAGCTCGAATTCTAGTATCCATTATCTTTAG |
