# Supplementary material for: Mutation of 4-coumarate: coenzyme A ligase 1 gene affects lignin biosynthesis and increases the cell wall digestibility in maize brown midrib5 mutants
Source: Biotechnol Biofuels. 2019 Apr 10;12:82. doi: 10.1186/s13068-019-1421-z (PMC6456989; doi:10.1186/s13068-019-1421-z)
Supplement: Supplementary file 5 — Additional file 5: Fig. S2. The 4CL activity of soluble protein extracts containing Zm4CL1 and the truncated Zm4CL1 mutants. [file 13068_2019_1421_MOESM5_ESM.docx]

**Additional file 5: Fig. S2** The 4CL activity of soluble protein extracts containing Zm4CL1 and the truncated Zm4CL1 mutants. Crude proteins extracted from lysate of *E. coli* cultures containing pET32a empty vector, pET32a-Zm4CL1, pET32a-Zm4CL1-L, and pET32a-Zm4CL1-S vectors were employed for 4CL activity assay.
