# Supplementary material for: Mutation of 4-coumarate: coenzyme A ligase 1 gene affects lignin biosynthesis and increases the cell wall digestibility in maize brown midrib5 mutants
Source: Biotechnol Biofuels. 2019 Apr 10;12:82. doi: 10.1186/s13068-019-1421-z (PMC6456989; doi:10.1186/s13068-019-1421-z)
Supplement: Supplementary file 6 — Additional file 6: Fig. S3. Phylogenetic analysis of 4CLs in vascular plants. [file 13068_2019_1421_MOESM6_ESM.docx]

**Additional file 6: Fig. S3** Phylogenetic analysis of 4CLs in vascular plants.

A maximum likelihood tree was constructed in Mega version 5.0 on the basis of multiple alignments of the deduced protein sequences from seven species. Sequence data used can be downloaded from Phytozome under the following accession numbers: *Medicago truncatula* Medtr2g105570, Medtr4g005750, Medtr4g128337, Medtr5g007640; *Arabidopsis thaliana* At1g51680, At3g21240, AT3g21230, At1g65060; *Zea mays* GRMZM2G048522, GRMZM2G054013, GRMZM2G055320, GRMZM2G075333, GRMZM2G174732; *Populus trichocarpa* Potri.001G036900, Potri.003G188500, Potri.006G169600, Potri.006G169700, Potri.018G094200, Potri.019G049500; *Oryza sativa* LOC_Os02g08100, LOC_Os02g46970, LOC_Os06g44620, LOC_Os08g34790; *Sorghum bicolor* Sb04g005210, Sb04g031010, Sb07g007810, Sb07g022040, Sb10g026130; *Saccharum* spp. hybrids Sh4CL1 (ANR02565), Sh4CL2 (ANR02566); *Panicum virgatum* Pv4CL1 (ACD02135), Pv4CL2 (ADZ96250).
