# Supplementary material for: Mutation of 4-coumarate: coenzyme A ligase 1 gene affects lignin biosynthesis and increases the cell wall digestibility in maize brown midrib5 mutants
Source: Biotechnol Biofuels. 2019 Apr 10;12:82. doi: 10.1186/s13068-019-1421-z (PMC6456989; doi:10.1186/s13068-019-1421-z)
Supplement: Supplementary file 7 — Additional file 7: Table S4. Expression levels of Zm4CL genes in maize different tissues. [file 13068_2019_1421_MOESM7_ESM.docx]

**Additional file 7: Table S4.** Expression levels of *Zm4CL* genes in maize different tissues.

| Tissues | Genes | | | | |
| --- | --- | --- | --- | --- | --- |
|  | *GRMZM2G174732* | *GRMZM2G075333* | *GRMZM2G055320* | *GRMZM2G048522* | *GRMZM2G054013* |
| root_system_7DAS | 67.5 | 1363.4 | 660.3 | 13.7 | 72.4 |
| internode_0DAP | 58.4 | 1919.6 | 134.4 | 0 | 35.5 |
| internode_6DAP | 60.2 | 486.7 | 34.1 | 0 | 108.2 |
| internode_12DAP | 64.8 | 704.3 | 311.9 | 0 | 23 |
| internode_18DAP | 91.8 | 851.9 | 21.4 | 0 | 28.9 |
| internode_24DAP | 80.8 | 505.9 | 15.9 | 0 | 21.5 |
| internode_30DAP | 100.2 | 447.9 | 17.3 | 0 | 19.4 |
| leave_0DAP | 0 | 196.2 | 0 | 0 | 204.2 |
| leave_6DAP | 23.8 | 265.3 | 8.5 | 22.8 | 63 |
| leave_12DAP | 12.5 | 212 | 13.1 | 44.5 | 117.3 |
| leave_18DAP | 21.2 | 309 | 13.7 | 59.2 | 97.5 |
| leave_24DAP | 48.3 | 258.4 | 10.3 | 111.1 | 137.7 |
| leave_30DAP | 50.5 | 470.2 | 16.3 | 212.2 | 59.9 |
| immature tassel | 0.3 | 45.2 | 78.4 | 0 | 70.3 |
| meiotic tassel | 20 | 1164.8 | 1368.1 | 0 | 42.1 |
| anthers | 14.6 | 332.9 | 632.2 | 0 | 46.8 |
| immature cob | 0 | 21.5 | 37.9 | 0 | 64 |
| pre-pollination cob | 0.9 | 28.9 | 138.2 | 0 | 35.1 |
| silk | 6.4 | 479.3 | 970.3 | 0 | 367.3 |
| whole_seed_2DAP | 2.5 | 65.8 | 240.1 | 0 | 50.1 |
| whole_seed_6DAP | 7.7 | 185.6 | 433.4 | 0 | 85.9 |
| whole_seed_10DAP | 37.9 | 357.6 | 823 | 0 | 47.7 |
| whole_seed_14DAP | 37 | 154.4 | 314.7 | 0 | 41.7 |
| whole_seed_18DAP | 52.3 | 117.2 | 267.3 | 0 | 40.5 |
| whole_seed_22DAP | 45.3 | 76.6 | 219.6 | 0 | 35.1 |
