# Supplementary material for: Mutation of 4-coumarate: coenzyme A ligase 1 gene affects lignin biosynthesis and increases the cell wall digestibility in maize brown midrib5 mutants
Source: Biotechnol Biofuels. 2019 Apr 10;12:82. doi: 10.1186/s13068-019-1421-z (PMC6456989; doi:10.1186/s13068-019-1421-z)
Supplement: Supplementary file 8 — Additional file 8: Fig. S4. Transcriptome analysis of bm5 mutant by microarray. [file 13068_2019_1421_MOESM8_ESM.docx]

**Additional file 8: Fig. S4** Transcriptome analysis of *bm5* mutant by microarray.

Gene ontology (GO) enrichment analysis of differentially expressed genes between *bm5*-504J mutant and B73 wild type maize. BP, Biological Process; MF, Molecular Function; CC, Cellular Component.
