# Supplementary material for: Mutation of 4-coumarate: coenzyme A ligase 1 gene affects lignin biosynthesis and increases the cell wall digestibility in maize brown midrib5 mutants
Source: Biotechnol Biofuels. 2019 Apr 10;12:82. doi: 10.1186/s13068-019-1421-z (PMC6456989; doi:10.1186/s13068-019-1421-z)
Supplement: Supplementary file 9 — Additional file 9: Table S5. Signal intension of the probe sets of regulated genes in bm5-504J mutant and B73 wild-type plant. [file 13068_2019_1421_MOESM9_ESM.docx]

**Additional file 9: Table S5.** Signal intension of the probe sets of regulated genes in *bm5*-504J mutant and B73 wild type plant.

| Probe Set ID | *bm5*-504J/Control | Function |
| --- | --- | --- |
| Zm.2766.1.A1_at | 7.02 | peroxidase |
| Zm.1085.1.A1_a_at | 2.38 | chitinase |
| Zm.16805.8.S1_at | 2.12 | chitinase |
| Zm.813.1.S1_at | 2.83 | glutathione *S*-transferase |
| Zm.7493.1.A1_at | 7.41 | alpha-mannosidase |
| Zm.5025.1.A1_at | 4.68 | alpha-mannosidase |
| Zm.3808.1.S1_at | 2.37 | defense response |
| Zm.1967.1.A1_at | 4.73 | defense response |
| Zm.672.1.A1_at | 2.07 | beta-expansin 4 |
| Zm.16583.1.A1_at | 2.47 | hydrolase |
| Zm.14398.1.A1_at | 2.35 | methyltransferase |
| Zm.18054.1.A1_a_at | 34.96 | *O*-methyltransferase-like protein |
| Zm.18054.1.A1_at | 5.74 | *O*-methyltransferase-like protein |
| Zm.529.1.A1_at | 2.33 | homocysteine *S*-methyltransferase-2 |
| Zm.9019.1.A1_at | 3.91 | serine/threonine kinase |
| Zm.1413.1.S1_at | 2.23 | receptor-like protein kinase |
| Zm.9034.1.A1_at | 4.73 | serine-type endopeptidase inhibitor |
| Zm.7023.1.S1_at | 3.14 | lipid metabolic process |
| Zm.6367.1.S1_at | 0.32 | 1-aminocyclopropane-1-carboxylate synthase |
| Zm.18501.1.A1_at | 0.34 | xyloglucan endo-1,4-beta-D-glucanase |
| Zm.1527.2.A1_a_at | 0.34 | xyloglucan:xyloglucosyl transferase |
| Zm.13338.1.S1_at | 0.44 | serine O-acetyltransferase |
| Zm.17087.1.S1_at | 0.05 | lipid transfer protein |
| Zm.10136.1.A1_at | 0.19 | CCR4-associated factor 1-like protein |
| Zm.1871.1.A1_at | 0.22 | embryo-abundant protein |
| Zm.14973.1.A1_at | 0.46 | acetylglucosaminyltransferase |
| Zm.9425.2.A1_x_at | 0.39 | prolin-rich protein |
| Zm.9425.1.A1_a_at | 0.37 | prolin-rich protein |
| Zm.16538.1.S1_at | 0.45 | ABC transporter |
| Zm.6393.1.A1_x_at | 0.08 | H1 histone |
| Zm.14513.5.S1_at | 0.37 | histone H3.3 |
| Zm.18132.1.S1_at | 0.24 | I-box binding factor - like protein |
| Zm.6393.2.S1_x_at | 0.07 | H1 histone |
| Zm.13245.1.S1_at | 0.41 | DREB1A |
| Zm.10842.1.S1_a_at | 0.47 | DRE binding factor |
| Zm.7734.1.A1_at | 0.28 | WRKY transcription factor |
| Zm.8637.1.A1_at | 0.18 | C2H2-type zinc finger protein |
| Zm.12139.1.A1_at | 0.35 | caltractin |
| Zm.11809.1.A1_at | 0.19 | phi-1-like phosphate-induced protein |
| Zm.18290.1.S1_at | 0.42 | serine/threonine phosphatase |
| Zm.6344.1.A1_at | 0.43 | serine/threonine phosphatase |
| Zm.9768.1.A1_at | 0.36 | receptor-like serinethreonine kinase |
| Zm.8610.1.A1_at | 0.41 | carbonyl reductase |
| Zm.12208.1.A1_at | 0.36 | cytochrome P450 |
| Zm.4035.1.A1_at | 0.26 | threonine kinase |
| Zm.17245.1.S1_at | 0.34 | serine/threonine kinase |
| Zm.5225.1.A1_at | 0.50 | protein kinase |
| Zm.19134.1.A1_at | 0.49 | oligopeptide transporter-like protein |
| Zm.11658.1.A1_at | 0.24 | calmodulin-related protein |
| Zm.39.1.S1_s_at | 0.46 | calcium-dependent protein kinase |
| Zm.11848.1.A1_at | 0.49 | calcium-binding protein |
| Zm.9197.2.A1_at | 0.42 | calcium-binding protein |
| Zm.16733.1.A1_at | 0.32 | calcium-transporting ATPase 1 |
| Zm.15937.1.A1_at | 0.29 | cytokinin oxidase-like protein |
| Zm.19356.1.A1_at | 0.13 | IAA-amino acid hydrolase |
| Zm.283.1.S1_at | 0.15 | indole synthase |
| Zm.7611.1.A1_a_at | 0.37 | Auxin-responsive protein |
