# Supplementary material for: Mutation of 4-coumarate: coenzyme A ligase 1 gene affects lignin biosynthesis and increases the cell wall digestibility in maize brown midrib5 mutants
Source: Biotechnol Biofuels. 2019 Apr 10;12:82. doi: 10.1186/s13068-019-1421-z (PMC6456989; doi:10.1186/s13068-019-1421-z)
Supplement: Supplementary file 10 — Additional file 10: Table S6. Signal intension of the probe sets of lignin genes in bm5-504J mutants and B73 wild-type plants. [file 13068_2019_1421_MOESM10_ESM.docx]

**Additional file 10: Table S6.** Signal intension of the probe sets of lignin genes in *bm5*-504J mutant and B73 wild type plant.

| Genes | Probes | *bm5*-504J/Control |
| --- | --- | --- |
| *PAL* | Zm.15903.1.S1_at | 0.90 |
|  | Zm.15903.2.A1_at | 0.94 |
|  | Zm.15903.3.A1_at | 1.15 |
| *C4H* | Zm.7134.1.S1_at | 1.96 |
| *4CL* | Zm.1390.1.A1_at | 0.68 |
|  | Zm.11845.1.A1_at | 1.17 |
|  | Zm.3406.1.S1_at | 0.58 |
| *CCR* | Zm.86.1.A1_at | 0.74 |
| *C3H* | Zm.4274.1.S1_at | 1.26 |
|  | Zm.10178.1.A1_at | 1.11 |
| *COMT* | Zm.1502.1.A1_at | 1.10 |
|  | Zm.13929.1.S1_at | 1.66 |
| *CCOMT* | Zm.3098.1.A1_at | 1.22 |
|  | Zm.233.1.S1_s_at | 1.30 |
| *CAD* | Zm.64.1.A1_at | 1.13 |
|  | Zm.17627.1.A1_at | 1.22 |
| *HCT* | Zm.1508.1.A1_at | 0.96 |
|  | Zm.18229.1.A1_at | 0.50 |
|  | Zm.5509.1.A1_at | 0.87 |
|  | Zm.16074.1.A1_at | 0.85 |
