# Supplementary material for: Mutation of 4-coumarate: coenzyme A ligase 1 gene affects lignin biosynthesis and increases the cell wall digestibility in maize brown midrib5 mutants
Source: Biotechnol Biofuels. 2019 Apr 10;12:82. doi: 10.1186/s13068-019-1421-z (PMC6456989; doi:10.1186/s13068-019-1421-z)
Supplement: Supplementary file 11 — Additional file 11: Fig. S5. Expression levels of other 4CL paralogs in bm5 mutants. [file 13068_2019_1421_MOESM11_ESM.docx]

**Additional file 11: Fig. S5** Expression levels of other *4CL* paralogs in *bm5* mutants.
