# Supplementary material for: Mutation of 4-coumarate: coenzyme A ligase 1 gene affects lignin biosynthesis and increases the cell wall digestibility in maize brown midrib5 mutants
Source: Biotechnol Biofuels. 2019 Apr 10;12:82. doi: 10.1186/s13068-019-1421-z (PMC6456989; doi:10.1186/s13068-019-1421-z)
Supplement: Supplementary file 12 — Additional file 12: Table S7. Lignin content and composition of bm5 mutant. [file 13068_2019_1421_MOESM12_ESM.docx]

**Additional file 12: Table S7.** Lignin content of *bm5* mutant.

| Plants^§^ | AcBr lignin  (mg.g^-1^ CWR) | Lignin monomer composition (μmol.g^-1^ CWR) | | | | S/G |
| --- | --- | --- | --- | --- | --- | --- |
|  |  | *p*-hydroxyphenyl (H) | Guaiacyl (G) | Syringyl (S) | H+G+S |  |
| B73 | 210.2±7.7 | 11.40±0.11 | 159.4±1.7 | 92.5±4.6 | 263.4±6.4 | 0.580±0.022 |
| *bm5* | 207.8±3.7 | 14.12±0.07** | 67.4±3.7** | 99.1±4.3 | 180.6±8.1** | 1.472±0.016** |

^§^ Midribs of the 2nd to 5th leaves from the top were collected from 60-d old *bm5*-504J mutant and B73 wild type maize. Values are means ± SE (n=3). One or two asterisks indicate significance corresponding to P < 0.05 or 0.01 (Student’s t-test).
