# Supplementary material for: Mutation of 4-coumarate: coenzyme A ligase 1 gene affects lignin biosynthesis and increases the cell wall digestibility in maize brown midrib5 mutants
Source: Biotechnol Biofuels. 2019 Apr 10;12:82. doi: 10.1186/s13068-019-1421-z (PMC6456989; doi:10.1186/s13068-019-1421-z)
Supplement: Supplementary file 13 — Additional file 13: Table S8. LC–PDA–ESI-MS/MS identification of soluble phenolics in methanolic extracts from midribs of the bm5 mutant. [file 13068_2019_1421_MOESM13_ESM.docx]

**Additional file 13: Table S8.** LC-PDA-ESI-MS/MS identification of soluble phenolics in methanolic extracts from midribs of the *bm5* mutant.

|  | | Peak 1 | Peak 2 | Feruloyl glucoside (FG) |
| --- | --- | --- | --- | --- |
| Retention time (min) | | 12.6 | 14.0 | 12.6 |
| UV λmax (nm) | | 320 (sh) | 320 (sh) | 320 (sh) |
| Negative MS/MS | Parent ions (*m/z*) | 367 [M-H]^-^ | 355 [M-H]^-^ | 355 [M-H]^-^ |
|  | Daughter ions (*m/z*) | 193 [ M-H-174]^-^  149 [ M-H-218]^-^ | 193 [M-H-162]^-^  175 [M-H-162-18]^-^ | 193 [ M-H-162]^-^ |
| Deduced compound | | Feruloyl quinic acid (FQA) | Feruloyl glucoside (FG) | - |
