# Supplementary material for: Mutation of 4-coumarate: coenzyme A ligase 1 gene affects lignin biosynthesis and increases the cell wall digestibility in maize brown midrib5 mutants
Source: Biotechnol Biofuels. 2019 Apr 10;12:82. doi: 10.1186/s13068-019-1421-z (PMC6456989; doi:10.1186/s13068-019-1421-z)
Supplement: Supplementary file 14 — Additional file 14: Fig. S6. SDS-PAGE analysis of recombinant AtUGT84A1, ZmUGT84A-1, and UGT84A-2 proteins. [file 13068_2019_1421_MOESM14_ESM.docx]

**Additional file 14: Fig. S6** SDS-PAGE analysis of recombinant AtUGT84A1, ZmUGT84A-1, and UGT84A-2 proteins. S and I indicate soluble and insoluble proteins from lysate of *E. coli* cultures containing pET32a empty vector, pET32a- AtUGT84A1, pET32a- ZmUGT84A-1, and pET32a- ZmUGT84A-2 vectors. The arrows show the recombinant proteins.
