# Supplementary material for: Mutation of 4-coumarate: coenzyme A ligase 1 gene affects lignin biosynthesis and increases the cell wall digestibility in maize brown midrib5 mutants
Source: Biotechnol Biofuels. 2019 Apr 10;12:82. doi: 10.1186/s13068-019-1421-z (PMC6456989; doi:10.1186/s13068-019-1421-z)
Supplement: Supplementary file 15 — Additional file 15: Fig. S7. The characterization of UDP-glucoside transferase forming glucose ester with ferulate in vitro. [file 13068_2019_1421_MOESM15_ESM.docx]

**Additional file 15: Fig. S7** The characterization of UDP-glucoside transferase forming glucose ester with ferulate *in vitro*. **a.** The profile of soluble phenolics in methanolic extracts from midribs of *bm5*-504J mutant. **b-d.** The formation of FG by adding ferulate and UDP-glucose into the crude extractive protein of recombinant proteins of AtUGT84A1 (b), ZmUGT84A-1 (c) and ZmUGT84A-2 (d). The arrow indicates FG. FG, feruloyl glucoside.
