# Supplementary material for: Mutation of 4-coumarate: coenzyme A ligase 1 gene affects lignin biosynthesis and increases the cell wall digestibility in maize brown midrib5 mutants
Source: Biotechnol Biofuels. 2019 Apr 10;12:82. doi: 10.1186/s13068-019-1421-z (PMC6456989; doi:10.1186/s13068-019-1421-z)
Supplement: Supplementary file 16 — Additional file 16: Fig. S8. The biosynthesis and characterization of FG and FQA in vitro. [file 13068_2019_1421_MOESM16_ESM.docx]

**Additional file 16: Fig. S8** The biosynthesis and characterization of FG and FQA in vitro. **a.** HPLC analysis of the purified FG. **b.** UV-Visible spectrum of the purified FG. **c.** Mass spectrum of the purified FG. **d.** HPLC analysis of the purified FQA. **e.** UV-Visible spectrum of the purified FQA. **f.** Mass spectrum of the purified FQA. The FG was prepared by using the production of the identified AtUGT84A1 toward ferulate and UDP-glucose. FQA was synthesized from FG and quinic acid after incubation with the crude extractive proteins from midribs of *bm5* mutant. FG, feruloyl glucoside. FQA, feruloyl quinic acid.
