# Supplementary material for: Mutation of 4-coumarate: coenzyme A ligase 1 gene affects lignin biosynthesis and increases the cell wall digestibility in maize brown midrib5 mutants
Source: Biotechnol Biofuels. 2019 Apr 10;12:82. doi: 10.1186/s13068-019-1421-z (PMC6456989; doi:10.1186/s13068-019-1421-z)
Supplement: Supplementary file 17 — Additional file 17: Fig. S9. UV–visible spectra of the remarkably accumulated products formed after adding FG into the crude extractive proteins from B73 and bm5-504J mutant. [file 13068_2019_1421_MOESM17_ESM.docx]

**Additional file 17: Fig. S9** UV-Visible spectra of the remarkably accumulated products formed after adding FG into the crude extractive proteins from B73 and *bm5*-504J mutant. **a.** UV-Visible spectrum of the small peak formed after adding FG into the boiled crude proteins extracted from B73 as a negative control in Fig. 6a. **b.** UV-Visible spectrum of FQA formed after adding FG into the crude extractive protein from B73 plant. **c.** UV-Visible spectrum of the small peak formed after adding FG into the boiled crude proteins extracted from *bm5*-504J mutant as a negative control in Fig. 6b. **d.** UV-Visible spectrum of FQA formed after adding FG into the crude extractive protein from *bm5*-504J mutant. Rt, retention time.
