# Supplementary material for: Mutation of 4-coumarate: coenzyme A ligase 1 gene affects lignin biosynthesis and increases the cell wall digestibility in maize brown midrib5 mutants
Source: Biotechnol Biofuels. 2019 Apr 10;12:82. doi: 10.1186/s13068-019-1421-z (PMC6456989; doi:10.1186/s13068-019-1421-z)
Supplement: Supplementary file 18 — Additional file 18: Table S9. ADL, hemicellulose, and cellulose contents of bm5 mutants and B73 wild-type plants. [file 13068_2019_1421_MOESM18_ESM.docx]

**Additional file 18: Table S9.** ADL, hemicellulose and cellulose contents of *bm5* mutants and B73 wild type plants.

| Plants^§^ | ADL (%CWR) | Hemicellulose (%CWR) | Cellulose(%CWR) |
| --- | --- | --- | --- |
| B73 | 5.93±0.152 | 20.98±0.87 | 18.75±0.93 |
| *bm5* | 4.36±0.077 ** | 19.89±1.33 | 20.40±2.31 |

^§^ Stalk samples were collected from 90-d old *bm5*-504J mutant and B73 wild type plants. Values are means ± SE (n=3).
